# Characteristics of Patients with Newly Diagnosed High-Grade Advanced Ovarian, Fallopian Tube, and Primary Peritoneal Cancer in 2018–2023 and the Impact of Molecular Diagnostics on Chemotherapy in Clinical Practice

**DOI:** 10.3390/biomedicines13020483

**Published:** 2025-02-15

**Authors:** Sonja Millert-Kalińska, Małgorzata Stawicka-Niełacna, Lidia Tomczak, Dominik Pruski, Marcin Przybylski, Karolina Jaszczyńska-Nowinka, Grzegorz Poprawski, Radosław Mądry

**Affiliations:** 1 Doctoral School, Poznan University of Medical Sciences, 61-701 Poznań, Poland; 2 District Public Hospital, Juraszów 7-19, 60-479 Poznań, Poland; lt.merlle@gmail.com (L.T.); dominik.pruski@icloud.com (D.P.); nicramp@poczta.onet.pl (M.P.); 3 Oncological Gynaecology Department, Poznan University of Medical Sciences, 60-569 Poznań, Poland; m.stawicka-nielacna@inm.uz.zgora.pl (M.S.-N.); karolina.jaszczynska-nowinka@usk.poznan.pl (K.J.-N.); grzegorz.poprawski@usk.poznan.pl (G.P.); radoslaw.madry@usk.poznan.pl (R.M.); 4 Department of Clinical Genetics and Pathology, University of Zielona Góra, Licealna 9, 65-046 Zielona Góra, Poland

**Keywords:** high-grade advanced ovarian cancer, OC, molecular diagnostics, HGAOC

## Abstract

**Background**: High-grade advanced ovarian, fallopian tube, and primary peritoneal (HGAOC) cancers require both surgical and systemic treatment. The introduction of polyADP-ribose polymerase inhibitors (PARPis) has significantly improved outcomes. This study presents an analysis of HGAOC patients treated at a single center, following updated guidelines. **Methods**: We observed 437 women newly diagnosed with HGAOC at the Department of Gynecological Oncology between January 2018 and December 2023. **Results**: Since November 2022, first-line treatment has included bevacizumab and PARPi, regardless of residual disease post-cytoreductive surgery. In both *BRCA1/2*-mutated and non-mutated groups, PARPi-based regimens increased significantly after May 2021 (*p* < 0.01). Recurrence number emerged as a strong prognostic factor for survival (*p* < 0.001), with each recurrence raising mortality risk by 80%. Median survival was 21 months for paclitaxel + platinum derivatives (PC), 27 months for PC + bevacizumab (BEV), and 30 months for PC + BEV + PARPi. **Conclusions**: The rapid adoption of modern therapies at our center has aligned treatment strategies with HRD status and global standards. However, variations in financial regulations and drug accessibility persist across countries. Despite these challenges, physicians should prioritize the most effective therapies available.

## 1. Introduction

Ovarian cancer (OC) typically presents in an advanced stage and ranks as the second leading cause of death among gynecological malignancies in women following cervical cancer. According to GLOBOCAN, projections indicate that in 2020, there were more than 300,000 newly reported cases of ovarian cancer, with over 200,000 deaths confirmed [1,2]. High-grade advanced ovarian, fallopian tube, and primary peritoneal cancer are malignancies with similar origin and histological structure; therefore, they are treated as one group of tumors, and the treatment regimens are common to them. Ovarian cancer is a cancer in which, apart from surgical treatment, systemic treatment plays an important role. The improvement in treatment results observed for several decades is associated with the introduction of platinum derivates, paclitaxel, bevacizumab, and polyADP-ribose polymerase inhibitors (PARPi).

In 1996, scientists presented the results of the GOG111 study, which proved higher effectiveness of OC treatment with cisplatin and paclitaxel compared with the previously used regimen, i.e., cisplatin combined with cyclophosphamide [3] in the group of patients with high-grade ovarian cancer (stage III and IV according to FIGO). Further results of the AGO-OVAR3 and GOG158 studies from 2003 revealed that carboplatin has similar effectiveness and a better tolerance profile to cisplatin and improves the quality of life [4]. The study results on bevacizumab, a recombinant humanized monoclonal antibody produced by recombinant DNA in the primary treatment of ovarian cancer, were initially published in 2010 by Burger R. et al. as a result GOG 218 trial [5]. The results of the ICON 7 (first time presented in 2010 and finally in 2015) study made it possible to define the group with the highest risk of disease progression from among the entire study group [6]. The “high-risk” group was defined as patients with the following characteristics: (1) stage III, undergoing suboptimal surgery (residual disease after surgery > 1 cm); (2) all patients with stage IV; (3) unoperated patients with stage III. However, it was the results of the GOG-0218 trial that formed the basis for introducing bevacizumab in Europe [7]. Median OS for stage IV bevacizumab-concurrent plus maintenance was 42.8 vs.32.6 months for stage IV control, but the differences were not statistically significant.

Previously, the introduction of a third chemotherapy drug resulted in more side effects while not improving the overall response rate (ORR) or prolonging progression-free survival (PFS) and overall survival (OS) [8,9,10]. The introduction of bevacizumab, which in the ICON 7 study showed higher ORR and prolongation OS, was not associated with the exposure of patients to aggravating side effects and did not negatively affect the quality of life [11,12].

Over the years, treatment of first-line OC has been associated with an increase in the response rate to treatment. Hence, more and more research has been conducted on consolidation and supportive therapy. Neither classic chemotherapy—based on paclitaxel with platinum derivatives, intraperitoneal chemotherapy, nor radio- nor immunotherapy proved sufficiently effective [13,14]. A real breakthrough in advanced ovarian, fallopian tube, and primary peritoneal cancer therapy was introduced by PARPis, which has revolutionized the treatment landscape for this disease [15]. The introduction of PARPis extended the time to disease progression, and this is the key element of first-line therapy.

Changes in the implementation of new therapies are inextricably linked to the introduction of new molecular diagnostic methods, which should be a two-stage cascade test. In the first stage, a patient with ovarian cancer is tested for the presence of *BRCA1/2* pathogenic or likely-pathogenic variants using Next Generation Sequencing (NGS) from cancer tissue. It is possible to determine not only germline mutation but also a somatic mutation in the tumors. Confirmation of the presence of *BRCA1/2* pathogenic or likely-pathogenic variants completes molecular diagnostics at this stage. However, if the patient is not a carrier of *BRCA1/2* pathogenic or likely-pathogenic variants, she should be tested for homologous recombination disorders. Testing for a homologous recombination deficiency (HRD) in primary high-grade ovarian cancer is crucial for recommending the appropriate therapy. Patients with a *BRCA* germline pathogenic or likely-pathogenic variant are defined as harboring an HRD (30%). However, there is a group of approximately 20% of patients harboring an HRD based on tumor genomic analyses.

PARPi were initially tested in patients with germline *BRCA1/2* (*gBRCA1/2*) pathogenic or likely-pathogenic variants [16,17,18]. In platinum-sensitive recurrent HGSOC, the response rate to PARPi monotherapy was approximately 30–45% higher among *BRCA1/2* pathogenic or likely-pathogenic variant carriers [19]. Subsequently, clinical trials pivoted their focus from PARPi monotherapy towards maintenance treatment post-platinum chemotherapy response, aiming to prolong the time to progression. In 2018, for the first time, the results of the SOLO 1 study were published in a group of patients with FIGO III and IV ovarian cancer with *gBRCA1/2* pathogenic or likely-pathogenic variants, who achieved a complete or partial response to treatment in the first-line of treatment [20]. The results of the SOLO 1 study were so groundbreaking that the drug olaparib was registered in Europe w 2019. New trials presented at the ESMO 2019 meeting indicate that patients without *BRCA1/2* pathogenic or likely-pathogenic variants also benefit from PARP inhibitors. The PRIMA trial was the first to evaluate the use of the PARPi, niraparib, in patients with advanced OC, regardless of BRCA mutation status and with residual disease. Niraparib, administered as monotherapy following first-line platinum chemotherapy, significantly improved progression-free survival (PFS) [21]. The PAOLA-1 study covered all patients with OC, but the beneficial effect of the use of platinum derivatives with bevacizumab and olaparib was proved only in a group of patients with advanced HGSOC with a damaged homologous recombination system (HRD [22]. Olaparib, in the PAOLA-1 schedule, was registered as a first-line treatment for OC in 2019. In November 2020, niraparib was registered in Europe—a new and only maintenance monotherapy after first-line chemotherapy for patients with advanced ovarian cancer, regardless of the *BRCA1/2* status. However, it was the results of the Chinese PRIME trial that contributed to expanding the indications for the use of niraparib, regardless of residual status after cytoreductive surgery [23].

The introduction of PARPi into clinical practice has opened a new chapter in the treatment of patients with ovarian cancer. It has offered hope for increasing the rate of long-term survival. The conditions for including patients in drug programs change over time, and the introduction of modern therapy is also accompanied by modern molecular diagnostics. Therefore, the importance of molecular tests should be emphasized.

**Figure 1:**
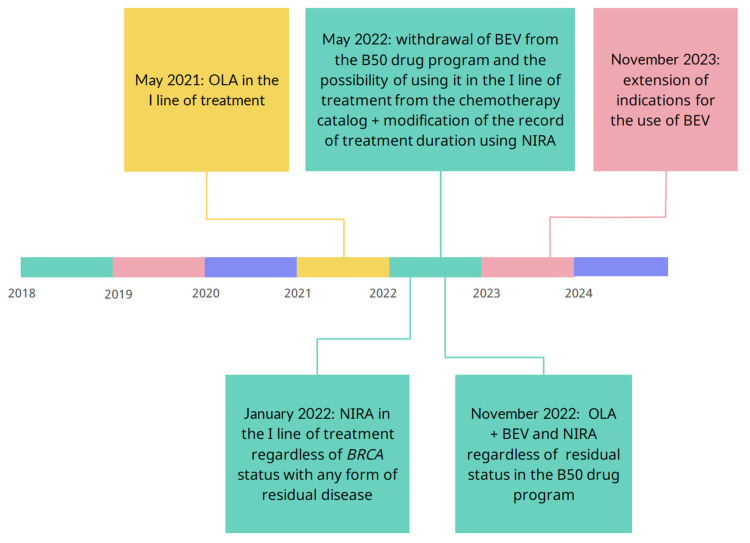
Analyzed periods in correlation with changes in the therapeutic program in Poland. OLA—olaparib; BEV—bevacizumab; NIRA—niraparib.

The criteria for inclusion in modern therapies in OC and the availability of them have changed dramatically in recent years, and along with changes in the world, we have witnessed their reflection in Polish conditions [24]. The subsequent points marked on the timeline in Figure 1 indicate the dynamic changes that have occurred in Polish health care in recent years. At that time Polish program was changed according to the PRIME trial results to use niraparib regardless of residual disease.

These modifications prompted the authors to show their impact on patients’ access to treatment. The aim of this study is to present a cross-section of patients treated for ovarian, fallopian tube, and primary peritoneal cancer in one center, including the distribution of treatment principles following the most up-to-date guidelines and considering the criteria for inclusion in drug programs.

## 2. Materials and Methods

This study received a positive opinion from the Poznań University of Medical Sciences’s Bioethics Committee of the lack of a medical experiment and its retrospective nature. We present a prospective observational study conducted in the University Clinical Hospital in Poznań (former Hospital of the Transfiguration of Lord, former Heliodor Święcicki Clinical Hospital), Poland, in 2018–2023.

### 2.1. Study Design

We observed patients who reported to the Department of Gyneacological Oncology between January 2018 and December 2023. We analyzed 437 women with a first diagnosis of ovarian, fallopian tube, and primary peritoneal cancer who had not been treated before. The inclusion criteria for the analysis were:

(1) high-grade ovarian, primary peritoneal, and fallopian tube cancer,
(2) serous histological type,
(3) FIGO stage III, IV.

The exclusion criteria in the analysis were:

(1) lack of chemotherapy due to the patient’s overall poor clinical condition or refusal of treatment,
(2) participation in clinical trials using another chemotherapeutic, e.g., pembrolizumab,
(3) incomplete documentation, including the non-staging type of OC.

**Scheme 1:**
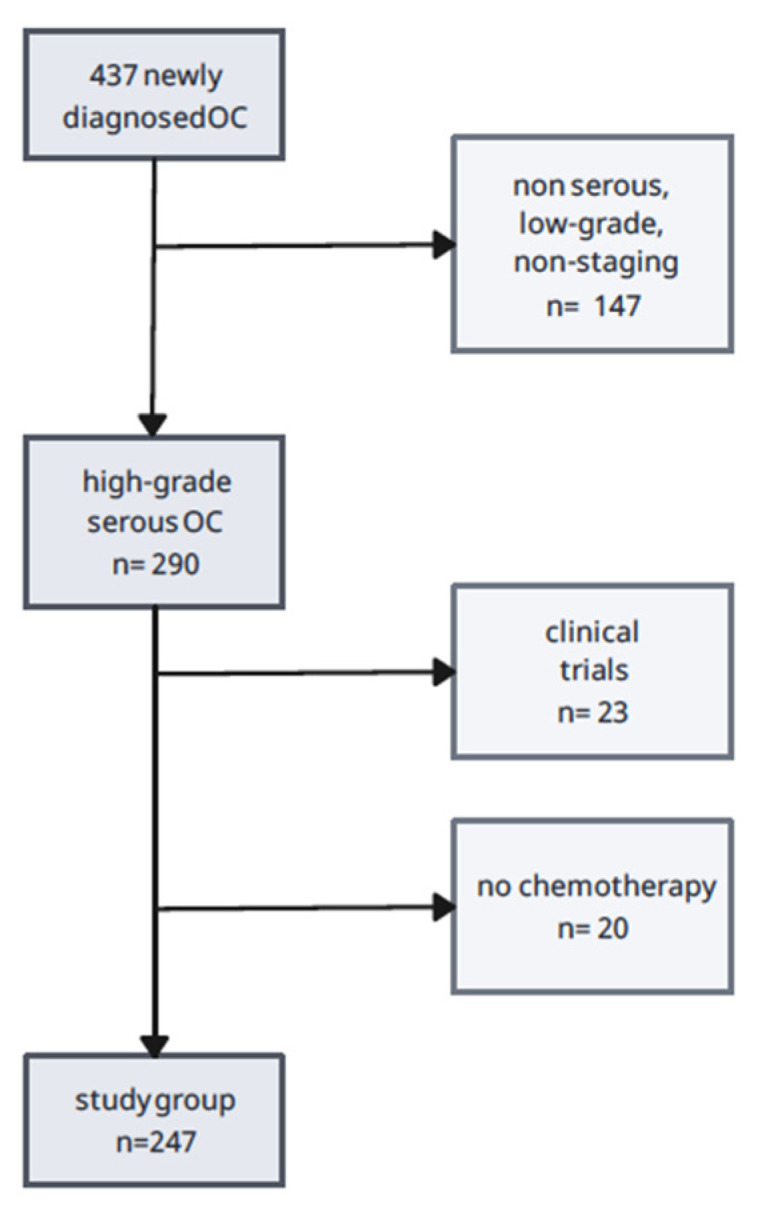
Study design considering inclusion and exclusion criteria. OC—ovarian cancer, primary peritoneal and fallopian tube cancer; n—number.

The study group is a real group of patients who were administered to the department for diagnostics or therapy, not a selected group of patients. Scheme 1 presents the subsequent stages of excluding individual patient cohorts so that the study group is as homogeneous as possible. Further divisions also included variables regarding the results of molecular tests that determined inclusion, in the absence of contraindications, in modern therapies using PARPi. For ease of description, patient groups will be marked BRCAmut, which means patients with *BRCA1/2* pathogenic or likely-pathogenic variants. Similarly, the patient group nonBRCAmut means patients with nonpathogenic or no clinical significance variant.

The above work analyzes and divides patients into groups regarding the chemotherapy drugs they take. It is worth noting that during the study period, both new therapeutic programs appeared, and the inclusion criteria have changed. The availability of PARP inhibitors changed in subsequent years, and the scope of use of bevacizumab has extended. As far as the scope of molecular analysis is concerned, all patients diagnosed at the level of genome sequencing from cancer tissue are cared for in a genetic clinic. The NGS method assessed the *BRCA1* and *BRCA2* genes with flanking sequences without assessing large rearrangements. Secondarily, patients at the clinic are also tested for other founder mutations—including mutations in the *PALB* or *CHECK* genes. Additionally, if the patient’s interview shows that ovarian cancer may be part of a genetic syndrome, e.g., Lynch syndrome, additional molecular diagnostics are also implemented.

### 2.2. Statistical Analysis

We conducted all analyses in the statistical software R, version 4.2.1 (R Core Team 2022, R: language and environment for statistical computing by the R Foundation for Statistical Computing, Vienna, Austria), with the *p*-value set at 0.05. Nominal variables are presented in the tables as the number and percentage of observations. Dependences between two variables were analyzed using a chi-squared test or Fisher’s exact test, depending on whether the assumption of expected counts of not less than five in at least 80% of the cells in the respective contingency table was satisfied. The strength of significant relationships between two categorical variables was measured using Cramer’s V coefficient.

## 3. Results

In total, two hundred forty-seven women met the inclusion and exclusion criteria. All patients were taking at least the paclitaxel + platinum derivatives regimen. Of the 247 subjects, 145 patients were treated with bevacizumab, which constitutes 58.7% of the study group. As far as PARPi is concerned, 79 patients used this treatment. Molecular analysis revealed the presence of *BRCA1/2* pathogenic or likely-pathogenic variants in 70 patients, which constitutes 28.3%. Molecular tests were not performed in 14.0%. Over study time, the number of molecular tests performed shows an increasing trend. Subsequently, in 2018, when molecular diagnostics were not commonly performed, tests for *BRCA1/2* pathogenic or likely-pathogenic variants were performed in 48.3% of patients, in 2019—67.9%, in 2020—91.1%, in 2021—97.8%, in 2022—96.4% and 2023—in 90.9% of patients.

Due to the changes in the availability of treatment such as PARPi and indications for first-line treatment, the treatment regimens were divided into three periods. Therefore, the first analyzed period is January 2018–April 2021. In May 2021, the possibility of treatment with olaparib in the first line of chemotherapy was introduced. The second time frame is November 2022 due to the introduction of changes to the list of reimbursed drugs. More patients may use modern treatment for ovarian cancer with combination therapy of PC with bevacizumab and PARPi, regardless of the remnants left during cytoreductive surgery. The reference points are shown in Figure 1. Table 1 shows the distribution of individual chemotherapy protocols used in patients in given periods.

The basic treatment regimen of paclitaxel with platinum derivatives (carboplatin or cisplatin) was received by 60 women, constituting less than ¼ of the study group (24.3%). It is noteworthy that 43/60 patients receiving this basic chemotherapy started their treatment between January 2018 and April 2021. Each patient with a presence of *BRCA1/2* pathogenic or likely-pathogenic variants admitted after May 2021 had the opportunity to use more modern therapy. As of May 2021, no carrier of *BRCA1/2* pathogenic or likely-pathogenic variants received paclitaxel + platinum derivates alone. The group of women receiving platinum derivatives with bevacizumab is 100, which constitutes 40.5% of the study group, but considering the combination of basic chemotherapy with bevacizumab and PARPi, 145/247 patients (58.7%) achieved it. The introduction of combined treatment with bevacizumab and PARPi into the first line of treatment, regardless of any remnants left during cytoreductive surgery, was introduced in November 2022. Since then, 30% (18/60) of patients (14 with olaparib and 4 with niraparib) have used the above regimen. Previously, from May 2021–October 2022, it was 27% of patients (13/81 with olaparib and 9/81 with niraparib). Among patients starting treatment in January 2018–April 2021, only 4.7% (5/106) received the platinum derivates + paclitaxel + olaparib regimen.

A detailed division of chemotherapy used in the groups of patients divided according to *BRCA1/2* pathogenic or likely-pathogenic status is shown in Table 2. The share of PARPi, particularly olaparib, in individual groups and the following timeframes is noteworthy. The platinum derivates + paclitaxel + bevacizumab + PARPi regimen in *BRCA1/2* pathogenic or likely-pathogenic variant carriers was used in 4.7% of patients (5/106) between January 2018 and April 2021 and in 16.0% of women (13/81) admitted between May 2021 and October 2022. Among patients admitted after November 1, 2022, 18/60 patients used the combined treatment of PC + bevacizumab and PARPi, which constitutes 30% of the study group. From this group, 11 were pathogenic or likely-pathogenic *BRCA1/2* variant carriers and additionally, seven women who were not BRCA mutation carriers might benefit from PARP inhibitors.

The following relationship is also noteworthy: in both carriers and not-carriers of *BRCA1/2* pathogenic or likely-pathogenic variants, significantly more patients used PC + PARPi or PC + BEV + PARPi in Periods II and III than in Period I (*p* < 0.01). Until April 2021, only about 30% of carriers of BRCA1/2 pathogenic or likely-pathogenic variants had access to PARP inhibitors, and in the following periods, 92.2% and 85.0%, respectively. These changes are also significant in patients from non-carriers of *BRCA1/2* pathogenic or likely-pathogenic variants; the increase in access to PARPi is 40-fold compared with Period I.

We also analyzed survival times in a group of patients treated with PARP inhibitors. Of the group of 93 patients, no recurrence was observed in 66–64 patients are still alive and 2 died. One recurrence occurred in thirteen patients (with a time to progression of 303–1348 days), and eight of them are still alive. Seven patients experienced two recurrences (with progression-free survival ranging from 43 to 509 days), and four of them are still alive. The group with three (with a progression-free survival period of 85 to 280 days) and four recurrences (from PFS 85–100 days) consisted of three patients each, and one from each group is still alive. A single patient who five recurrences, and she is no longer alive (the last PFS lasted 39 days). Among the patients who experienced recurrence during follow-up, their survival times can be presented:

- 25% of patients from the analyzed group died within 27 months (exactly 27.11 months) from the beginning of observation. This means that one-quarter of the patients had a survival time of less than 27 months.
- 50th percentile (median): 50% of patients died within 38 months (37.81 months, to be exact). The median means that half of the patients survived less than 38 months, and the other half lived longer.
- 75th percentile: 75% of patients died within 44 months (43.89 months, to be exact). This means that three-quarters of patients had a survival time of less than 44 months, and only 25% of patients survived beyond this period.

**Figure 2:**
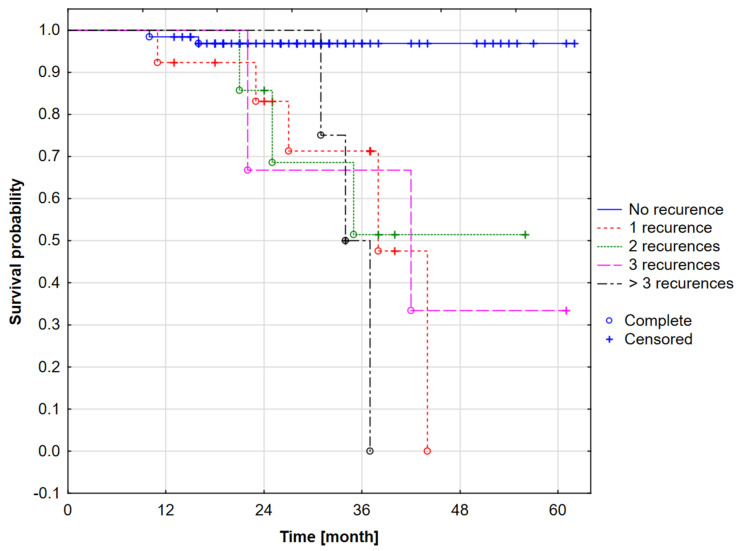
Survival probability of patients treated with PARPi depends on the number of recurrences.

**Figure 3:**
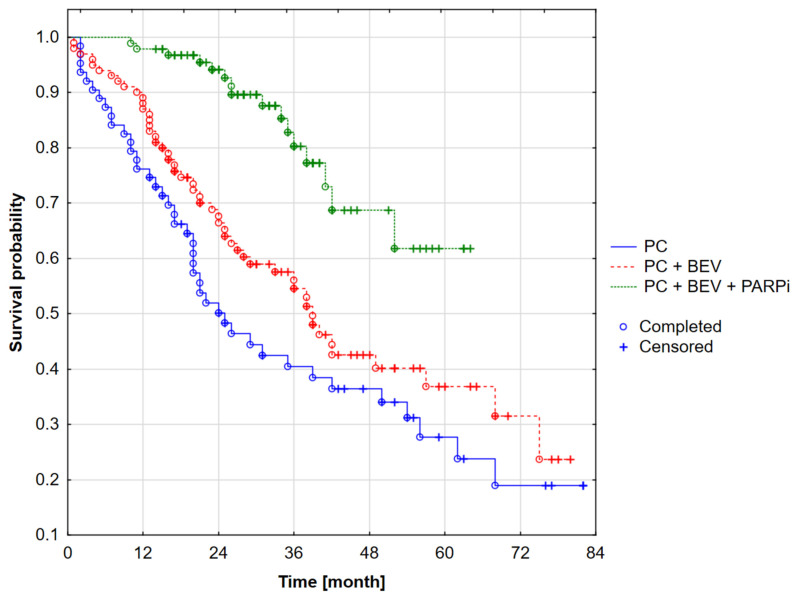
Survival times of patients treated with different chemotherapy regimens.

Figure 2 shows the Kaplan–Meier curves with a division into groups of patients with different numbers of recurrences. Due to the small number of patients with four recurrences (n = 3) and five recurrences (n = 1), these groups were merged. Figure 3 shows Kaplan–Meier curves for patients treated with different chemotherapy regimens. Survival analysis showed a statistically significant difference between the groups (log-rank test, *p* < 0.001). The shortest median survival was 21 months in the group treated with paclitaxel + platinum derivates (PC), while for the PC + BEV group, the median was 27 months. The longest median of 30 months was recorded in the group treated with combined treatment, including paclitaxel + platinum derivates with or without bevacizumab and PARP inhibitors (PC + BEV + PARPi).

The results indicate that the number of recurrences is a strong and statistically significant prognostic factor for survival (*p* < 0.001). Each additional recurrence increases the risk of death by 80%. The confidence interval (1.33–2.43) indicates the high precision of the estimate.

We also analyzed patients who had contraindications to bevacizumab. In total, 16 patients could not receive it, which is 6.5% of the study group. Of those patients, the majority of patients (9/16) could not receive bevacizumab because of failure to meet the criteria for inclusion in the drug program in the form of hematological disorders, neutropenia, or thrombocytopenia, 3 patients due to poor general clinical condition or disease progression, 3 patients due to a history of pulmonary embolism or cardiac contraindications, and 1 patient due to a non-healing postoperative wound.

## 4. Discussion

The above work aimed to show how changes in the availability of new therapeutic options and changing indications for the use of specific drugs affect the qualifications of patients and their use of modern therapy in advanced ovarian, fallopian tube, and primary peritoneal cancer. The changes in recommendations and drug usage allowed us to additionally answer the question about the value of molecular diagnostics, which is an indispensable element in introducing subsequent patients to therapeutic programs.

Since the Ministry of Health in Poland launched the B50 drug program in March 2014, updates have been systematically appearing in the form of annexes to the drug program.

Figure 1 presents the periods we analyze in relation to changes in the therapeutic program in Poland. Our analysis covers the last six years, so we have been following the patient since 2018. The analyzed period is divided into Period I (January 2018–April 2021), Period II (May 2021–October 2022), and Period III (November 2022–December 2023). A real revolution started with the implementation of PAPRP in advanced ovarian, fallopian tube, and primary peritoneal cancer. The analysis revealed that in Period I only, significantly more patients used PC or the PC + BEV combination before new therapeutic options became available. In the group of carriers of *BRCA1/2* pathogenic or likely-pathogenic variants, 24% of them received chemotherapy based on platinum derivatives with paclitaxel in Period I, and in the following years, none of them received chemotherapy alone. This means that every patient with OC FIGO III and IV who is a carrier of *BRCA1/2* pathogenic or likely-pathogenic variants had either PARPi as a maintenance treatment or received BEV alone. Patients not receiving bevacizumab most likely underwent radical cytoreductive surgery. However, the group receiving only chemotherapy with bevacizumab in Period I (44%) significantly decreased in favor of the introduction of PARP inhibitors, and this shift is visible in Table 1. In relation to patients with no *BRCA1/2* pathogenic or likely-pathogenic variants, niraparib has been present in therapy since the beginning of Period II, which indirectly proves that HRD patients also had the opportunity to use PARPis. The use of a combination of chemotherapy with BEV and PARPi in carriers of *BRCA1/2* pathogenic or likely-pathogenic variants in Periods II and III compared with Period I (61.9% and 50.0% vs. 17.2%) is statistically significant. The appearance of a provision on the use of niraparib in patients regardless of *BRCA* status is reflected in the availability of this drug—in Period I, no patients without *BRCA1/2* pathogenic or likely-pathogenic variants received it, while in Period II—18.6% and Period III—13.3%. Therefore, the introduction of changes in the availability of modern therapy in Poland very quickly changed the structure of patient treatment in our center.

Our study demonstrates a correlation between the changing availability of modern chemotherapy and the increasing number of patients who have access to it. Currently, as of July 2024, the highest usage of the treatment is achieved by patients who took in the first line of treatment—a combination of paclitaxel with platinum derivatives, bevacizumab, and a PARP inhibitor. Our results indicate that in the period until May 2021, 5/106 patients used such therapy, which constitutes 4.7% of patients starting their treatment in January 2018. In the period from May 2021 to October 2022, 22/81 patients took combined therapy, which constituted 27% of patients admitted to the department at that time. Since the official introduction of combination therapy in November 2022, we have observed an even greater increase in the number of patients with access to treatment—they accounted for 30% of the group (18/60). The authors assume that extending the observation period until 2024 might allow for even wider usage of PARPis. Therefore, such analysis and long-term follow-up must be performed to describe the effect of combined therapy on the prolongation of PFS.

Observing the changes that occurred for carriers of *BRCA1/2* pathogenic or likely-pathogenic variants between Period II and Period III, we can see that by October 2022, 95% of patients had contact with PARPi, and from November 2022, 85% of patients had. This slight shift may first indicate that the patients are autonomous and decide about their treatment. Noteworthy, in Period I, as many as 2/3 of *BRCA1/2* pathogenic or likely-pathogenic variant carriers could not have access to PARPi. This means that the introduction of public financing of PARP inhibitors in Poland has dramatically changed the fate of patients, at least those with *BRCA1/2* pathogenic or likely-pathogenic variants.

Our analysis confirmed that for personalized and patient-oriented treatment, genetics diagnostics must be performed as soon as possible. Molecular tests were performed in almost 85% of patients in our study. It is known that patients who are not *BRCA1/2* pathogenic or likely-pathogenic variant carriers but have a homologous recombination deficiency (HRD) benefit from additional treatment with PARP inhibitors [25]. Introducing them to therapy, thanks to new indications for reimbursement, opened the door and gave hope to many new patients. In Poland, although the need for molecular diagnostics has been paid more in recent years, there is no funding for HRD testing, hence the low percentage of results. Considering current recommendations (as of July 2024), only molecular diagnostics for *BRCA1/2* pathogenic or likely-pathogenic variants in patients diagnosed with OC are covered by reimbursement. According to data presented in the above work, such diagnostics in patients with a first diagnosis of advanced ovarian, fallopian tube, and primary peritoneal cancer in our center were performed by 85.4% of patients, with an increasing trend over time, but still does not reach 100% coverage of the population. We have not found any data from other foreign centers in the literature that would provide percentage coverage of the population with molecular diagnostics. However, other studies also emphasize the need to prioritize clinical trials of innovative treatment methods and improve predictive biomarkers that will enable the selection of patients who would benefit from targeted treatment [26].

At the very end of the work, which clearly indicates the benefits of keeping up with the times in medicine, there is an emphasis on the role of artificial intelligence (AI) in medicine. Machine learning (ML) has a substantial impact on healthcare and might continue to reshape this field. Enshaei A, Robson C, and Edmondson R in their study investigated the potential for an AI model to provide prognostic and predictive information and compared it with conventional statistical approaches for ovarian cancer, which is characterized by its heterogeneity; therefore, conventional algorithms become too complex for routine clinical use [27]. The potential in the field of oncology is tremendous and has applications in almost every aspect of cancer research, including diagnosis, prognosis, and treatment [28]. In the future, artificial intelligence AI and Ml will select the profile of molecular and immunohistochemical screening tests depending on the risk factors for cancer. Moreover, after obtaining the final histopathological diagnosis with a full molecular analysis of the tumor and genetic risk, he will select an appropriate drug program. The full use of AI and ML requires scientific confirmation and must remain under the strict supervision and control of doctors. Work on their application in many fields is ongoing, and the results presented so far are promising.

Our work is interesting in that very few centers present therapy regimens for their patients. We believe that sharing such knowledge would be an interesting voice in the discussion. It can be observed that many of our patients use combined therapy. Adding a PARP inhibitor to the combination of platinum derivatives + paclitaxel and bevacizumab used during chemotherapy is the most natural treatment as a continuation of the first line. The use of molecular diagnostics is crucial and should be performed as soon as possible after the histopathological diagnosis of cancer. Thanks to this, information about the molecular structure of cells can directly influence therapeutic decisions and the most personalized treatment.

Still, we lack direct data on whether PARPi alone is more valuable than PARP inhibitors used together with bevacizumab. Vergote I et al. assume that patients treated with PARP inhibitors combined with bevacizumab had better results than those treated with niraparib or olaparib alone [29]. However, it should be remembered that this study is indirect and should be treated with great caution.

The main limitation of our study is a single-center analysis and retrospective character of this study. Still, we decided to present it to encourage other clinicians to look at the structure of patient groups and share their experiences. To objectively assess the results of patients’ treatment with PARP inhibitors and thus estimate progression-free survival or overall survival, follow-up should be continued for several more years. Patient clinical data from the current work and their fate will be followed, and we plan to publish the treatment results soon.

This study is the first in Poland to show the profile of patients clearly and reliably with newly diagnosed advanced ovarian, fallopian tube, and primary peritoneal cancer. We assume that this is the first step aimed at sharing knowledge and transparency of the proposed treatment models and proof that more and more patients are using current therapies following the latest recommendations. We are aware of how comprehensive care oncological patients require, especially those who have advanced ovarian, fallopian tube, and primary peritoneal cancer [29,30,31]. This process is most effective and complete if performed immediately after a cancer diagnosis is obtained. The importance of patient education by health providers but also through social campaigns, should also be emphasized. Patients should be aware of diagnostic and therapeutic options and demand the best treatment for themselves, and we, as healthcare professionals, should treat our patients following current medical knowledge.

## 5. Conclusions

The aim of this study was to address changes in the treatment of ovarian cancer based on new molecular diagnostic tools, including genetic testing, which have become popular over the last decade. The introduction of modern treatment in our center very quickly changed the structure of patient treatment—based on the results of the presence of a pathogenic or probably pathogenic *BRCA* variant in the field of HRD. These results are consistent with global data and standards, but it should be noted depending on the country, funding sources and the patient’s medical history, chemotherapy varies. Analysis of the impact of these factors on therapy may significantly translate into overall survival and time to first recurrence. Extending the follow-up for another five years will enable final results and conclusions to be reached. However, all doctors should strive to offer their patients the most effective therapy possible.

## Figures and Tables

**Table 1:** Distribution of chemotherapy in patients in analyzed periods in correlation with changes in the therapeutic program.

		Period		I/January 2018–April 2021 (40 Months)			II/May 2021–October 2022 (17 Months)			III/November 2022–December 2023 (13 Months)		
				n	% of a Specific Group	% of the Group from the Whole Period	n	% of a Specific Group	% of the Group from the Whole Period	n	% of a Specific Group	% of the Group from the Whole Period
		BRCAmut										
PC	-	-	-	7	24.1%	6.6%	0	0.0%	0.0%	0	0.0%	0.0%
PC	BEV	-	-	13	44.8%	12.3%	1	4.8%	1.2%	3	15.0%	5.0%
PC	BEV	OLA	-	5	17.2%	4.7%	13	61.9%	16.0%	10	50.0%	16.7%
PC	BEV	-	NIR	0	0.0%	0.0%	0	0.0%	0.0%	1	5.0%	1.7%
PC	-	OLA	-	4	13.9%	3.8%	7	33.3%	8.7%	5	25.0%	8.3%
PC	-	-	NIR	0	0.0%	0.0%	0	0.0%	0.0%	1	5.0%	1.7%
				29	100.0%	27.4%	21	100.0%	25.9%	20	100.0%	33.4%
		nonBRCAmut										
PC	-	-	-	21	42.9%	19.9%	6	10.5%	7.4%	7	20.0%	11.7%
PC	BEV	-	-	27	55.1%	25.5%	27	47.4%	33.3%	13	37.1%	21.7%
PC	BEV	OLA	-	0	0.0%	0.0%	0	0.0%	0.0%	4	11.4%	6.6%
PC	BEV	-	NIR	0	0.0%	0.0%	9	15.8%	11.1%	3	8.6%	5.0%
PC	-	OLA	-	1	2.0%	0.9%	0	0.0%	0.0%	0	0.0%	0.0%
PC	-	-	NIR	0	0.0%	0.0%	15	26.3%	18.6%	8	22.9%	13.3%
				49	100.0%	46.3%	57	100.0%	70.4%	35	100.0%	58.3%
		no data										
PC	-	-	-	15	53.6%	14.1%	2	66.7%	2.5%	2	40.0%	3.3%
PC	BEV	-	-	12	42.9%	11.3%	1	33.3%	1.2%	3	60.0%	5.0%
PC	BEV	OLA	-	0	0.0%	0.0%	0	0.0%	0.0%	0	0.0%	0.0%
PC	BEV	-	NIR	0	0.0%	0.0%	0	0.0%	0.0%	0	0.0%	0.0%
PC	-	OLA	-	1	3.5%	0.9%	0	0.0%	0.0%	0	0.0%	0.0%
PC	-	-	NIR	0	0.0%	0.0%	0	0.0%	0.0%	0	0.0%	0.0%
				28	100.0%	26.3%	3	100.0%	3.7%	5	100.0%	8.3%
		total		106			81			60		

PC—paclitaxel with platinum derivates; BEV—bevacizumab; OLA—Olaparib; NIR—niraparib; n—number.

**Table 2:** Distribution of chemotherapy in patients in analyzed periods in correlation with changes in the therapeutic program according to *BRCA*mut status.

BRCA Status		Period I		Period II		Period III		All		χ^2^	*p*	V_c_
		N	%	N	%	N	%	N	%			
BRCAmut	PC	7	24.1%	0	0.0%	0	0.0%	7	10.0%	26.86 *	<0.001	0.45
	PC + BEV	13	44.8%	1	4.8%	3	15.0%	17	24.3%			
	PC + PARPi	4	13.8%	7	33.3%	6	30.0%	17	24.3%			
	PC + BEV + PARPi	5	17.3%	13	61.9%	11	55.0%	29	41.4%			
	all	29	100.0%	21	100.0%	20	100.0%	70	100.0%			
	PARPi together	9	31.0%	20	95.2%	17	85.0%					
	PC	21	42.9%	6	10.5%	7	20.0%	34	24.1%	36.13 *	<0.001	0.34
	PC + BEV	27	55.1%	27	47.4%	13	37.1%	67	47.5%			
non-BRCA-mut	PC + PARPi	1	2.0%	15	26.3%	8	22.9%	24	17.0%			
	PC + BEV + PARPi	0	0.0%	9	15.8%	7	20.0%	16	11.4%			
	all	49	100.0%	57	100.0%	35	100.0%	141	100.0%			
	PARPi together	1	2.0%	24	42.1%	15	42.9%					

N—number; χ^2^ chi-square test; *p*—*p*-value; V_c_—effect strength index; *—exact Fisher’s test; Period I: January 2018–April 2021; Period II: May 2021–October 2022; Period III: November 2022–December 2023; PC—paclitaxel and platinum derivates; BEV—bevacizumab; PARPi—poly ADP-ribose polymerase inhibitors.

## Data Availability

All data are available from the corresponding author.
